# Open-source Longitudinal Sleep Analysis From Accelerometer Data (DPSleep): Algorithm Development and Validation

**DOI:** 10.2196/29849

**Published:** 2021-10-06

**Authors:** Habiballah Rahimi-Eichi, Garth Coombs III, Constanza M Vidal Bustamante, Jukka-Pekka Onnela, Justin T Baker, Randy L Buckner

**Affiliations:** 1 Department of Psychology Harvard University Cambridge, MA United States; 2 Institute for Technology in Psychiatry McLean Hospital Belmont, MA United States; 3 Department of Psychiatry Harvard Medical School Boston, MA United States; 4 Department of Biostatistics Harvard T.H. Chan School of Public Health Harvard University Boston, MA United States; 5 Athinoula A. Martinos Center for Biomedical Imaging Massachusetts General Hospital Charlestown, MA United States

**Keywords:** actigraphy, accelerometer, sleep, deep-phenotyping, smartphone, mobile phone

## Abstract

**Background:**

Wearable devices are now widely available to collect continuous objective behavioral data from individuals and to measure sleep.

**Objective:**

This study aims to introduce a pipeline to infer sleep onset, duration, and quality from raw accelerometer data and then quantify the relationships between derived sleep metrics and other variables of interest.

**Methods:**

The pipeline released here for the deep phenotyping of sleep, as the *DPSleep* software package, uses a stepwise algorithm to detect missing data; within-individual, minute-based, spectral power percentiles of activity; and iterative, forward-and-backward–sliding windows to estimate the major Sleep Episode onset and offset. Software modules allow for manual quality control adjustment of the derived sleep features and correction for time zone changes. In this paper, we have illustrated the pipeline with data from participants studied for more than 200 days each.

**Results:**

Actigraphy-based measures of sleep duration were associated with self-reported sleep quality ratings. Simultaneous measures of smartphone use and GPS location data support the validity of the sleep timing inferences and reveal how phone measures of sleep timing can differ from actigraphy data.

**Conclusions:**

We discuss the use of DPSleep in relation to other available sleep estimation approaches and provide example use cases that include multi-dimensional, deep longitudinal phenotyping, extended measurement of dynamics associated with mental illness, and the possibility of combining wearable actigraphy and personal electronic device data (eg, smartphones and tablets) to measure individual differences across a wide range of behavioral variations in health and disease. A new open-source pipeline for deep phenotyping of sleep, DPSleep, analyzes raw accelerometer data from wearable devices and estimates sleep onset and offset while allowing for manual quality control adjustments.

## Introduction

### Background and Challenge

Prolonged daily episodes of sleep behavior are expressed nearly ubiquitously in all members of our species, as they are innate and undergird both physical and mental health across the lifespan. Multiple studies have suggested that sleep loss or poor sleep quality are predictors (and potentially moderators and mediators) of mental illness symptoms and poor cognitive performance [1-6]. While the use of modern digital devices influences sleep timing, these devices also afford new low-burden opportunities to measure sleep [7-10]. This paper introduces an open-source sleep-analysis pipeline called DPSleep, referring to the deep phenotyping of sleep that we offer to the community as a platform to facilitate longitudinal studies of sleep using data from widely available wearable devices [11].

The current gold standard for documenting sleep timing and content is polysomnography (PSG), during which multiple physiological measures are recorded, usually in a clinic or laboratory setting [12,13] or recently in ambulatory settings [14,15]. Although PSG is a comprehensive assessment of sleep stages, there are limitations associated with cost and subject burden, and it is difficult to obtain in-patient or at-home versions of PSG for extended periods [16,17]. Actigraphy, defined as recording activity-related data, mainly acceleration, using wearable devices, has been suggested as an efficient and reliable alternative to measure certain features of sleep patterns in natural, at-home settings [18,19]. Actigraphy data, estimated from accelerometers, are a common output of many wearable and held devices, including wrist watches, ankle bands and wristbands, smartglasses, sewn-in or attached devices, and smartphones [15,20-24]. At the same time, openly available feature-extraction algorithms with the capability to retain and present the features from raw to derived measures are essential for a reproducible large-scale understanding of human sleep, even in the presence of proprietary algorithms associated with many of the devices.

### Objective

In actigraphy-based sleep assessment using wristbands, acceleration is typically measured in 3 dimensions, where each axis—x, y, and z—reflects linear acceleration along with one dimension of a triaxial accelerometer; some devices also measure environmental factors such as ambient light, temperature, or physiological measures such as heart rate variability or electrodermal activity [25]. A body of literature investigating the sensitivity and specificity of wristbands to detect sleep parameters, including total sleep time, sleep onset latency, wake after sleep onset, and sleep efficiency, has evolved [26-28]. Although several of these approaches have been validated using PSG and self-reported sleep quality ratings under certain controlled conditions, there is ongoing research to validate the software and develop in-house algorithms for different applications and under real-world circumstances [29-31]. Several of the devices, in order to save memory and battery, provide preprocessed, 1-minute averaged acceleration data [6,32-35], whereas others provide continuous high-frequency data [36-38].

This study contributes to this evolving field by providing a comprehensive pipeline to analyze raw accelerometer data to estimate minute-based activity and detect the major Sleep Episode, defined as the longest continuous sleep episode of at least 100 minutes. The overall goals are to (1) develop an open-source processing pipeline to detect major Sleep Episodes from commonly available accelerometer data; (2) apply and validate the estimation procedure using real-world data, including individuals studied over extended periods who independently rated their sleep; and (3) apply the processing pipeline to exemplar data to illustrate its application. To this end, we aim to analyze data from undergraduate students who are studied over 6-9 months during college to illustrate sleep patterns that fluctuate with environmental demands and in relation to other self-report measures of sleep and mental state. We also aim to analyze data from 2 individuals who are outpatients with severe mental illness to illustrate the boundaries of the methods and their ability to measure dramatically altered sleep patterns.

## Methods

### Participants

Participants were enrolled into 2 distinct cohorts to obtain actigraphy data across a range of individuals. The samples are described separately.

#### Study 1: Undergraduate Study

In total, 6 undergraduate participants (all aged 19 years; 3 females; 4 White participants, 1 unspecified, and 1 Asian; all non-Hispanic) were recruited from a local private institution and participated for one academic year (165-268 days), including a buffer extending into the summer break. These individuals had successfully participated in a shorter, earlier pilot study that did not use the present actigraphy device or processing pipeline. Participants were compensated per hour for the lab visits and for completing daily app-based questionnaires and given milestone bonuses to encourage continued participation. Participants were required to be enrolled full-time in classes and own an iPhone or Android smartphone compatible with the study smartphone app, Beiwe, which is part of the open-source Beiwe platform for digital phenotyping [39]. The Beiwe app was configured to collect passive phone use, phone acceleration, and GPS data at an almost continuous rate, as well as active self-report data on a regular, daily basis. As each participant served as their own baseline, participants were not excluded for current or past psychiatric disorders or medication use. Mental health history was measured by self-report of current or past diagnoses of mental disorders, current or past use of prescribed psychotropic medications, and current or past concerns about mental health symptoms (undiagnosed). Only 1 participant reported any current or past mental health history (current psychotropic medication for anxiety). All study procedures were approved by the Institutional Review Board of Harvard University.

#### Study 2: Clinical Study

Two individuals (aged 62 and 24 years; 1 female) were recruited from an ongoing cohort following the clinical progression of severe mental illness at a local hospital. Individuals were diagnosed with psychotic disorders (bipolar, n=1; schizophrenia, n=1) using a structured clinical interview for Diagnostic and Statistical Manual of Mental Disorders (DSM)-IV [40]. The diagnoses were initially acquired before DSM-V gaining traction. However, the primary diagnosis was reviewed periodically, and DSM-V-(revised version) [41] criteria were referenced when adjusting the diagnoses. Participant enrollment for this study targeted obtaining >1 year of data for each participant (duration: 543 and 309 days), which included a nearly continuous collection of smartphone and actigraphy data via the Beiwe platform [39] and wearable watch, respectively, from each participant. Participants were compensated for these data and for monthly in-person study visits during which clinical assessments were recorded to quantify disease progression using clinical gold standard measures. Milestone bonuses were provided to encourage continued participation. All study procedures were approved by the Institutional Review Board of Partners Healthcare.

### Wrist Actigraphy and Ancillary Data Acquisition

The present pipeline was developed using triaxial acceleration data from a commercially available waterproof watch worn on the wrist (GENEActiv, Activinsights Ltd) and is intended as a general open resource for processing accelerometer data from any device that saves raw triaxial, high-frequency, continuous accelerometer data, sampled at a fixed and known rate. Missingness of data was assumed to occur completely at random. Data saved as minute-based or shorter activity estimates can also be accommodated. The frequency of data sampling was set to 30 Hz for study 1 and 20 Hz to preserve the memory in case the patients missed their study visits for study 2. Following the initial consent and receipt of the watch, individuals in studies 1 and 2 visited the lab every 4-5 weeks to return the watch and receive a new, fully charged watch with formatted memory; participants in study 2 were given the watches during their in-patient study visits. Participants were instructed to wear the watch continuously, including during sleep and while bathing. Using the same sampling rates across modalities of 30 Hz in study 1 and 20 Hz in study 2, the watch collected acceleration (g), light (lux), and ambient temperature (C). In addition, the wristband recorded key presses. Participants were instructed to press the key when they started to go to sleep and when they woke in the morning. The acceleration data are the primary data used for the automatic detection of episodes that would be scored as sleep with additional corrections from light and key press data when available and when necessary.

Data obtained from a smartphone (iPhone or Android) were used as an ancillary data source [39,42]. None of the automated processing or manual Sleep Episode adjustments used data from the smartphone. The smartphone data provided valuable independent information for validation. Individuals installed the research smartphone app, Beiwe, to collect active (questionnaire), passive phone use (via timestamping of lock-unlock events), accelerometer, and GPS data [39,43,44]. The GPS location of the phone was sampled every 10 minutes for a 2-minute duration, and phone acceleration was sampled 10 seconds on and 10 seconds off. At 5 PM each day, an in-app questionnaire appeared that asked about the quality of the previous night's sleep on a Likert scale (5-point Likert scale from 0 [exceptional] to 4 [terribly]), the amount of caffeine consumed in the previous 24 hours (5-point Likert scale from 0 [none] to 4 [five +]; Multimedia Appendix 1), and a set of questions about their mood and social and academic activities. Answers to these questions and passive estimates of phone use were used to validate the identification of the major Sleep Episodes from the independent watch actigraphy data.

### DPSleep: A Processing Pipeline for Deep Phenotyping of Sleep

#### Raw Actigraphy Data and Removal of Missing Data (Wrist-Off)

Raw wrist actigraphy data were originally saved as large, compressed files, each containing multiple weeks of data. Each file comprises a table with columns of acceleration, light, and temperature (depending on the device). To overcome the challenge of time-consuming access to specific rows during analysis, the first step in our processing pipeline is to parse and save the data into separate daily files from midnight to 11:59 PM. For days on which a watch change occurred (to allow continuous data sampling), a new, already charged watch was placed on the wrist. As the old watch keeps collecting nonwrist data until it is connected back to the data extraction station, the data from the new watch were formatted to overwrite any data from the previous watch at the same clock times.

Raw actigraphy data (30 Hz) are displayed for one daily data file illustrating each separate accelerometer trace (Figure S1 of Multimedia Appendix 2). This type of raw actigraphy data measures linear acceleration and is thus most sensitive to dynamic movements with varying amplitudes and frequencies during different activities such as walking, phone typing, or even tossing and turning in bed [45]. The amplitude of the fluctuations, measured in units of gravity (g), reflects the relative acceleration of the actigraphy device related to the gravity of the Earth. Each axis—x(g), y(g), and z(g)—reflects linear acceleration along with one dimension of a triaxial accelerometer. The raw data always include the Earth’s gravity component, which can be reflected on different axes depending on the orientation of the device. The separate channels are highly correlated and, for our purposes here, provide redundant information that can be interrogated separately or combined to make estimates more robust. The amplitude of the accelerometer fluctuations varies across the awake hours but shows a stark reduction in fluctuation in all three axes during the sleep period. As explained below, the DPSleep processing pipeline is optimized to detect the reduction in fluctuations and estimate a single extended episode each day.

**Figure 1:**
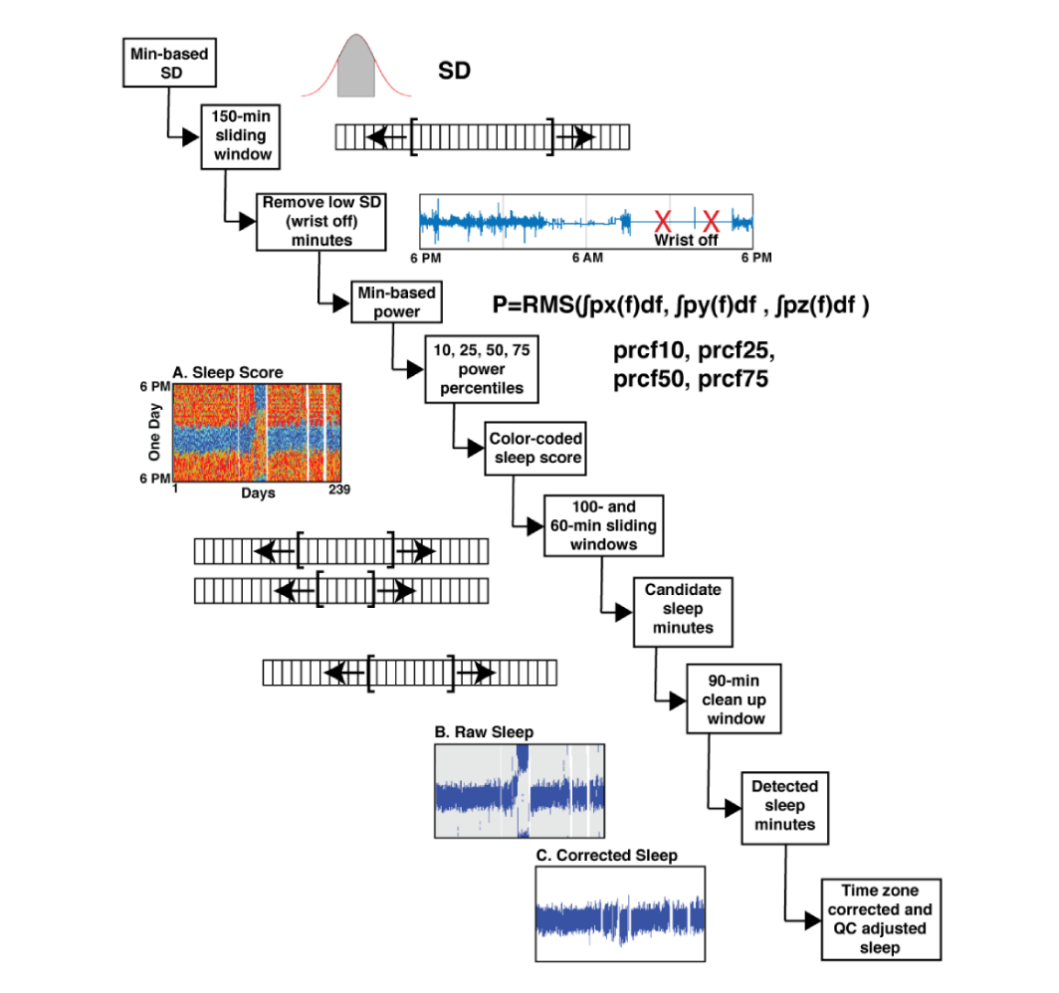
The DPSleep processing pipeline. The sequential steps in the processing pipeline are illustrated with example data for key steps. The pipeline begins with raw accelerometer data and derives estimates of individualized sleep scores (A) and raw (B) and corrected Sleep Episodes (C). Sleep variables are calculated based on the corrected Sleep Episodes as estimated in C. Specifically, as the first step, the SD of the acceleration along three axes is averaged over 150-minute windows forward and backward to find the wrist-off minutes. The power density spectrum of the acceleration signal is calculated at different frequencies, and the area under the curve estimates power as the root mean squared integrated over the three axes. Minutes are classified based on 10, 25, 50, and 75 percentile thresholds and can be visualized (A; blue, cyan, green, orange, and red display the minutes based on increasing power scores). A series of forward and backward moving average windows is used to flag the candidate Sleep Episodes that are then further filtered to derive the raw sleep estimate (B). Quality control and adjustment for the local time zone then yield to the final corrected sleep estimate (C). QC: quality control; RMS: root mean square.

An immediate challenge is that, even for compliant participants with charged waterproof actigraphy watches, they occasionally removed their devices. Figure 1 shows an epoch in which the watch was off the wrist. Unlike the Sleep Episode, which contains residual periodic low levels of fluctuations, the accelerometer traces are nearly flat during the wrist-off periods. The first step is to detect and remove the wrist-off minutes. As the focus of these analyses is to identify the major Sleep Episode, a window size of 150 minutes is used to detect the wrist-off minutes. The SD of the acceleration time series for each of the three axes was calculated for each minute. A forward-and-backward–moving window of SD for each axis was calculated for a window size of 150 minutes. Then, the root mean square (RMS) of the moving average SD values for the three axes is compared with a small experimental threshold of 0.0185 to detect the wrist-off minutes. This threshold was determined based on more than 600 hours (5 volunteers from our research group for 5-7 days each) of annotated data collected in-house. The minute of data is considered wrist-off if the RMS of the variance of the three channels is below this threshold. The remaining data were considered for further analyses.

#### Scoring Activity Level for Each Minute

After removing the wrist-off minutes from the analysis, the power density spectrum of the acceleration signal in each minute was calculated using Welch formula [46]. The power of the signal in each minute is the area under the curve of the power density spectrum. Within-individual power thresholds were then determined for 10, 25, 50, and 75 percentiles. A 15-minute-wide window of the average RMS power that combines across the three axes is used to find the percentile thresholds specific to each individual. Data from each minute of the study were classified based on their spectral power in comparison with these percentile cut-offs, and color-coded daily maps of the activity scores for each individual were generated (Figure 1, center-left). Highly active minutes are colored in red and orange, minutes with medium activity are colored in green, and low active minutes are colored in blue and cyan. Daily maps provide intuitive information about the sleep patterns of the individual. As the watch is recording continuous actigraphy without reference to the external world, when the time zone is changed due to traveling, the sleep pattern is shifted and will need to be accommodated at a later stage of processing, as can be seen in Figure 1 at approximately day 150. Standard or daylight saving time transitions require correction.

#### Estimating Major Sleep Episodes

To automatically estimate the timing of the Sleep Episode, we used multiple moving windows that slide over a weighted transformation of the minute-based activity levels. The minutes with less than 25% activity (25th percentile of the empirical distribution of activity for the person) are assigned a sleep score of 1, and those with higher than 50% activity are penalized by negative experimental scores (-0.75 and -1). Then, two moving windows of narrow (60 minutes) and wide (100 minutes) sizes were used to sweep the scores and find a provisional nocturnal episode of sleep, while the narrower window adjusts the beginning and end estimates of the major Sleep Episode. The separation of tasks between two window sizes was found, in pilot analyses, to better capture the distinct targeted events, where detecting the nocturnal Sleep Episode benefitted from the larger window, but the precision of the sleep onset and offset time estimates benefitted from the smaller window. A *clean-up* 90-minute wide moving window was then used to connect adjacent short candidate Sleep Episodes with more than three-quarter sleep-scored minutes in each 90-minute window. However, this process, on some occasions, left two separate candidate Sleep Episodes that were separated by a period of activity during the middle of the night. As a convention, the automated algorithm joined discontinuous Sleep Episodes into one longer episode if they fell within 22.5 minutes (a quarter of the 90-minute window) of one another. This is a decision of convention and occurred in 2.34% (34/1448) of the cases in which sleep was measured in healthy young adults.

The outcome of these steps is an estimate of a single provisional Sleep Episode for each day. The estimate, via the filtering approaches used, usually underestimates the full Sleep Episode duration by not including minutes on either temporal side of the sliding windows that have low activity. To mitigate this bias, as a final step, the initial estimate was expanded or shrunk to include all adjacent minutes that show less than 25% activity so long as they were after (when available) the evening button press (indicating the participant’s intended start of attempting to sleep) and before their waking button press. A button press was considered available if the device successfully recorded a button press within 60 minutes of the estimate. The compliance of the individuals to provide informative (within 60 minutes of the estimate) button presses for sleep or bed ranged from 76.9% to 90.4% across the 6 participants in study 1 (mean 84.0%, SD 5.5%) and was 0.3% and 22.6% for the 2 participants in study 2. The duration of the major Sleep Episode after these corrections is recorded in the data output files as the automatically generated *SleepDuration* with its beginning (*SleepOnset*) and end (*SleepOffset*) times.

Figure S2 of Multimedia Appendix 2 displays examples of the initial automatic estimate of the Sleep Episode (yellow bottom bar) and the final corrected Sleep Episode (middle green bar) in the third row of panels A, B, and C. A third estimate, shown as a blue bar, expands from the final Sleep Episode to include adjacent minutes when the activity is below the 50% threshold, such as when individuals are resting in bed but not yet asleep. We store the beginning of the expanded epoch as the *BedrestOnset*, the end as the *BedrestOffset*, and the duration as the *BedrestDuration.* For some purposes, the time from the beginning of the *BedrestOnset* to *SleepOnset* can be used as a distinct measure (eg, *SleepOnsetLatency*). The variables are listed in Textbox 1. The estimated light exposure level from the watch is also shown in Figure S2 of Multimedia Appendix 2, as well as the timestamps of the recorded button presses. The light level is not used by the algorithm to estimate the Sleep Episode but is visualized because it can aid manual adjustments that are applied during quality control. An assumption of the present approach is that a single Sleep Episode will occur that is usually 100 minutes or more; the limitations of this simplifying assumption will be discussed.

**Sleep variables generated by the DPSleep pipeline.**

**Parameter name and description**

- OffWrist: percentage of wrist-off time per 24 hours (%)
- SleepOnset: beginning of the Sleep Episode (hh:mm)
- SleepOffset: end of Sleep Episode (hh:mm)
- SleepDuration: difference between SleepOnset and SleepOffset (min)
- BedrestOnset: beginning of Bedrest Episode (hh:mm)
- BedrestOffset: end of Bedrest Episode (hh:mm)
- BedrestDuration: difference between BedrestOnset and BedrestOffset (min)
- SleepOnsetLatency: difference between BedrestOnset and SleepOnset (min)
- SleepEfficiency: percentage of SleepDuration or BedrestDuration (%)
- ActiveMinutes: number of minutes during Sleep Episode with activity higher than the 40th percentile
- ImmobileMinutes: number of minutes during Sleep Episode with activity lower than the 40th percentile
- ActiveBouts: number of bouts (sequences) during Sleep Episode with continuous ActiveMinutes, with 1-Min immobility tolerance
- SleepImmobility: percentage of immobile minutes or sleep duration (%)
- LightMinutes: number of minutes during Sleep Episode with light greater than 1 lux
- LightBouts: number of bouts during Sleep Episode with continuous LightMinutes, with 1-minute darkness tolerance
- PhoneMinutes: number of minutes during sleep episodes with any phone event, including locked, unlocked, or in use
- PhoneBouts: number of bouts during Sleep Episode with continuous PhoneMinutes, letting 1-minute no-event tolerance

### Smartphone Data

In addition to wearing the actigraphy watch, most of the individuals in studies 1 and 2 installed the Beiwe app on their smartphones. The Beiwe app was configured to passively collect phone on or off times and the GPS location of the phone. An independent pipeline was used to securely analyze the GPS data and extract the places most visited by the individual during the study and estimate their major locations every 12 minutes. Then, a daily map was color-coded based on the presence of the individual at those points of interest. The GPS map provides the time zone of the locations where the subject has visited and evidence of location stability or movement around the time of the major Sleep Episode.

When participants traveled across time zones, a challenge arose as a matter of practice: when the new time zone was behind the old time zone, the data shifted back and overwrote the previously recorded data, and when the new time zone was ahead of the old one, the data shifted forward, and there was a missing data gap. The time shifts occurring during the actual days of travel, especially when travel occurs by plane, are challenging to incorporate, and we considered these days as missing data with the days before and after being retained with data shifted to reflect the time zone experienced by the participant. The participants in study 1 traveled two to four times during the course of the study, whereas the participants in study 2 did not travel. A similar time-shifting issue occurs for standard or daylight saving time transitions. In these cases, the time is shifted forward or backward accordingly, and the two transition nights are considered as missing data. Alternate goals, such as estimating circadian rhythms, may be better served by analyzing the data in a continuous fashion and will be different from the present focus, where the discrete daily patterns require these practical adjustments.

The smartphone data from Beiwe also provided relevant data for validation, including accelerometer and phone use data (via the recorded lock-unlock events). The DPSleep pipeline allows the integration and visualization of smartphone data when available. To integrate accelerometer data for the validation purposes of this study, a simple SD analysis was applied to the phone acceleration along the x-axis as a representative of the acceleration score to recognize minutes of high movement. The distribution of the acceleration score in all minutes during the study for each individual was used to find minutes with greater than 75 and 90 percentile movement (normalized to the individual). These minutes were color-coded in yellow and red, respectively, contrasting the lower acceleration minutes in gray and plotted in relation to the daily Sleep Episode estimates (Figure S3 of Multimedia Appendix 2). In addition, the locked-unlocked events of the phone document when the phone is in use. Phone in use time was defined as the time between every consecutive unlocked-locked event lasting no more than 15 minutes. This is to soften the strong assumption of the phone being in use all the time after the unlocked event and before it is locked again. The daily map is then color-coded to show the locked-unlocked minutes in red and blue, respectively, in addition to in-use minutes in green (Figure S3 of Multimedia Appendix 2). These data are used in this study to build confidence in the DPSleep estimates of the major Sleep Episodes. They may also be useful for understanding the relationship between digital technology use and sleep patterns, for example, as might occur if individuals use their phone sporadically at night.

### Sleep Estimation Quality Control

DPSleep should not be expected to deliver a perfectly accurate output when operating in automatic mode. Several assumptions are made, and the structure of an individual sleep night can be complex. Instead, DPSleep provides an elaborative day-by-day report, examples of which appear in Figure S2 of Multimedia Appendix 2. The user can decide about the confidence of the estimated Sleep Episode and revise the results manually, if necessary. DPSleep includes editing tools. Every page of the report presents the data about one day from 6 PM on the previous day to 6 PM on the original day. The report also includes, when available, smartphone data, as illustrated in Figure S3 of Multimedia Appendix 2. The daily report is a significant help to the investigators to decide about and increase the precision of the sleep estimation results based on the availability and richness of the data.

All results in this paper have been quality-controlled by 2 individuals independently relying on only the watch actigraphy data and not any ancillary data from the smartphone to make modifications. Thus, the data are analyzed here, as would be from any typical study that only obtained watch actigraphy data. The guidelines used for quality control are described in Multimedia Appendix 3. Figure S4 of Multimedia Appendix 2 illustrates the plots of sleep duration across nights before and after manual adjustments for each of the 6 individuals in study 1. As shown, most nights show identical values before and after quality control, meaning no adjustment was required, whereas many others showed slight adjustments. For several nights, a large adjustment was required (eg, in Figure S4 P6 of Multimedia Appendix 2, there is an outlier value; in Figure S4 P5 of Multimedia Appendix 2 there are several values that were substantially corrected). The automated values showed a correlation with the final corrected values that ranged between r=0.92 and r=0.98. Figure S5 of Multimedia Appendix 2 illustrates examples of errors that require manual adjustments. Manual adjustments were made for 18.02% (261/1448) of the nights. As illustrated in Figure S4 of Multimedia Appendix 2, although approximately one in five nights were adjusted, most were small adjustments that would not impact most analyses. Approximately 9.05% (131/1448) of the nights were adjusted to change the major Sleep Episode estimate by greater than 1 hour.

### Data Security

Throughout the analysis, the pipeline is designed to handle the data securely without exposing any identifiable information. Days are reported as the days of the study relative to the individual’s consent days. We consider the GPS data identifiable not only when the actual coordinates are presented but also the patterns of the daily maps as presented with significant (for the individual) locations. Therefore, the pipeline was designed to work with the encrypted data and avoid saving any of the coordinates or maps as unencrypted scratch files. The final results are saved directly in an encrypted format.

### Within-Individual Statistical Modeling

To analyze the longitudinal association between actigraphy-based sleep estimation and self-reported data, a simple individual-level linear model was used with self-reported sleep quality as a predictor and actigraphy-based sleep duration as the outcome. This simple linear model was selected after using a mixed linear model analysis to account for the time and autocorrelation of the observations. The categorical day of the week was included as a covariate to account for weekly structure related to course schedules and weekday-weekend differences. Only data collected during the academic year were included (ie, fall and spring semesters, including exam periods and Thanksgiving and spring breaks, but excluding winter and summer breaks) to account for differences between the school year and extended school breaks. All analyses were conducted in R (R Foundation for Statistical Computing) using the *stats* package *lm()* function [47].

### Code Availability

The DPSleep pipeline software package is available [11] as an open-source package to be downloaded and used by the research community.

## Results

### Longitudinal Activity-Based Sleep Estimates Within Individuals

**Figure 2:**
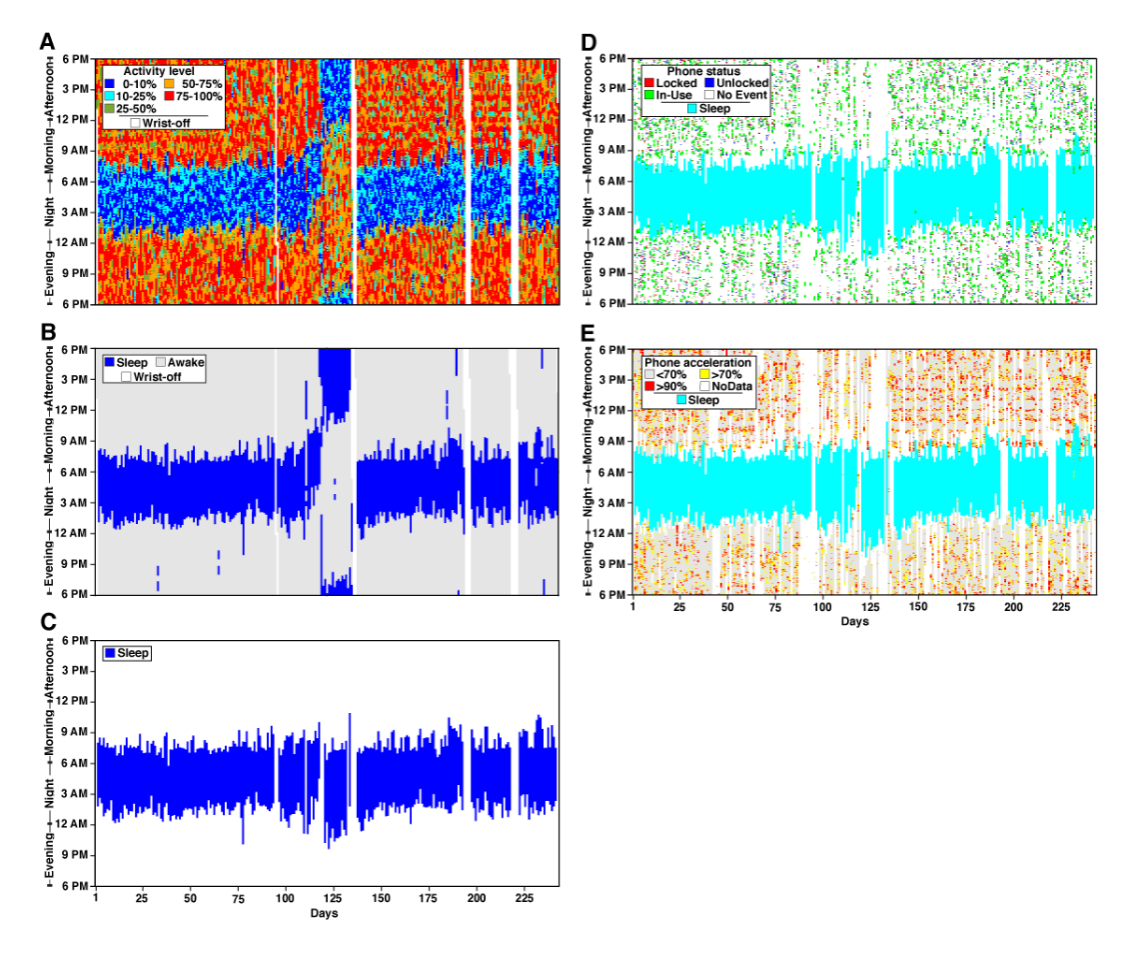
Longitudinal Sleep Episode estimates in P1 related to phone use. Three left panels display longitudinal activity score and Sleep Episode estimates for 243 days. Day 1 is near to the ninth day after the beginning of the semester. Winter break falls near days 102 to 133. Each vertical line displays data for a 24-hour day. (A) The top panel displays the continuous activity scores colored by the threshold for each day. For each day, the plot begins at 6 PM at the bottom and ends at 6 PM at the top, allowing the nighttime sleep period to plot in the center of the graph. Over days, the low activity band (in blue) is relatively consistent except for a dramatic shift at day 118, which is attributed to travel outside of the time zone. (B) The middle panel displays the same data revealing the automated detection of Sleep Episodes and nap periods. (C) The bottom panel C displays the time zone–adjusted and fully quality-controlled estimates of the final Sleep Episodes. On the right panels, the Sleep Episodes of panel C are plotted (light blue) in relation to independently estimated phone events. (D) The phone status is shown with colored hashing for when the phone is locked (red), unlocked (blue), and in use (green). Note that there is no phone use during the estimated Sleep Episodes. The phone use events, on many nights, occur up until and just before the beginning of the Sleep Episode. However, on most mornings, there is a gap between when the Sleep Episode ends and phone use begins, which often begins abruptly around 9 AM, possibly reflecting that phone use begins with an alarm-triggered event. (E) Phone acceleration data are plotted and also reveal no phone movement during the Sleep Episodes.

**Figure 3:**
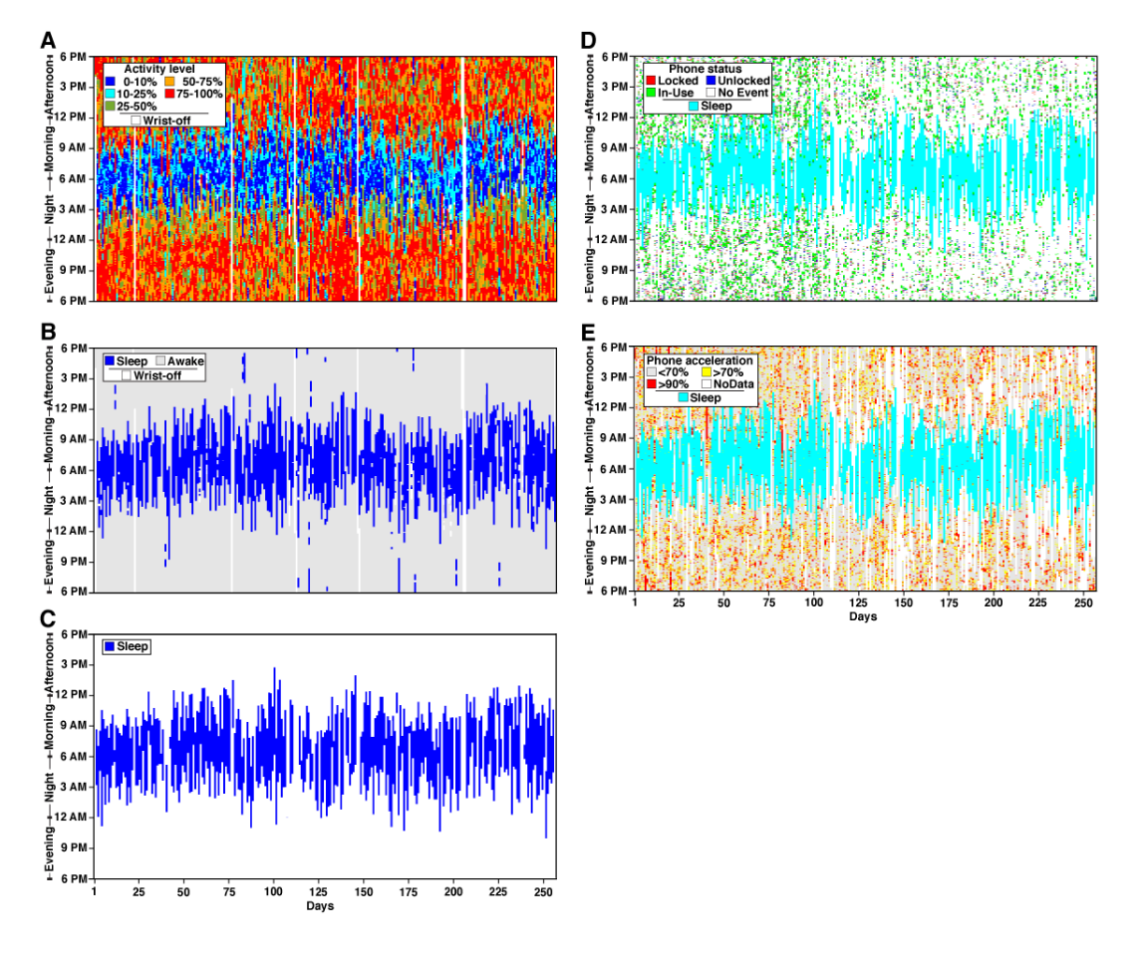
Longitudinal Sleep Episode estimates in P4 in relation to phone use. Longitudinal activity score, Sleep Episode estimates, and phone use data for 257 days are plotted. Day 2 is near to the beginning of the semester. Winter break falls near days 112 to 143. (A) The top-left panel shows the continuous activity scores colored by the threshold for each day. (B) The middle-left panel displays the same data revealing the automated detection of Sleep Episodes and nap periods. (C) The bottom-left panel demonstrates the time zone–adjusted and fully quality-controlled estimates of the final Sleep Episodes. On the right panels, the Sleep Episodes of panel C are plotted (light blue) in relation to independently estimated phone events. (D) The phone status is shown with colored hashing for when the phone is locked (red), unlocked (blue), and in use (green). (E) Phone acceleration data are plotted and also reveal no phone movement during the Sleep Episodes. This individual shows highly irregular Sleep Episodes. Note that the phone status and phone acceleration events track the irregular Sleep Episodes.

Longitudinal sleep patterns and daily sleep maps are the key outcomes of the DPSleep pipeline (Figure 1; see the *Methods* section). To show the performance of the pipeline, four examples of processed data are shown for individuals from study 1 (see the *Methods* section; Figures 2 and 3; Figure S6 and S7 of Multimedia Appendix 2). The data are from the full academic year with travel to and from campus and across daylight saving time. Each figure contains five plots, and three plots on the left are described here. Plot A is the color-coded daily map of activity scores that show the minutes with high (>50% orange; >75% red), low (<25% cyan; <10% blue), and medium (25%-50% green) activity. Plot B represents the raw estimated Sleep Episodes for each day. Plot C is the time zone–adjusted and quality control–adjusted longitudinal plot of sleep behavior. The third plot represents the data used for all the quantitative analyses and validations.

Figure 2 shows the sleep data for P1 with a highly regular sleep pattern where the subject goes to bed sometime between 1 AM and 2 AM and wakes up consistently near 8 AM. On weekends, the participant wakes up about an hour later than usual. Figure 3 shows the sleep data for P4, which displays the most irregular sleep patterns. There are some nights when this participant goes to bed at around midnight and does not wake up before 9 AM, and on other nights, the individual does not go to sleep before 6 AM and sleeps for only a few hours. The irregular sleep pattern does not appear to be related to the academic calendar because the participant displays a similar sleep pattern during breaks. In addition, the sleep data for P2 and P3 with a less regular sleep pattern compared with P1 are shown in Figure S6 and S7 of Multimedia Appendix 2. The effect of beginning the academic year is notable, with a gradual transition to a later sleep time (first 25 days). Break and travel also significantly affect the sleep schedule during days 125-150.

### Smartphone Use Tracks Sleep Episodes

One way of validating the estimated Sleep Episode arises from the independent phone data collected simultaneously through the Beiwe app on each individual’s cell phone. Even though these data do not continuously measure activity, as the phone can be put down, they do indicate the minutes during which the individual is clearly not sleeping. The right parts of Figures 2 and 3 and Figure S6 and S7 of Multimedia Appendix 2 plot the smartphone data for the 4 participants analyzed above. In each plot, panel D shows the phone status via locked-unlocked events. When the phone is not used, there should be no change in the phone status. Panel E displays phone acceleration that, similar to the wristwatch, provides a measure of dynamic movement when the phone is picked up, used, or is moving with the participant’s body.

In all participants, the phone activity measures were generally outside the time of the estimated Sleep Episodes and tracked the variations in sleep period from night to night. P2 and P3 (Figure S6 and S7 of Multimedia Appendix 2) show this quite clearly because their sleep patterns change gradually during the first 25 nights of measurement. Phone activity measures tracked these transitions. P4, who showed the most erratic sleep patterns, also demonstrated clear evidence that phone use was frequent and intensive only outside the time of the major Sleep Episodes (Figure 3). Beyond the general correspondence between phone and sleep, there were also interesting features in the details of the phone use that are relevant for comparing the activity- and phone-based data types.

As an additional visualization, Figure S8 of Multimedia Appendix 2 shows data from P1 with the horizontal axis representing clock time (48 consecutive hours, with the second 24 hours on each horizontal line repeated in the first 24 hours of the next horizontal line) and day number down the y-axis. This is the plotting convention often used by investigators interested in circadian rhythms [48]. DPSleep allows plotting using either lateral (as in most figures in this paper) or horizontal (eg, Figure S8 of Multimedia Appendix 2) conventions.

### Actigraphy Measures of Sleep Duration Track Self-report Sleep Quality

In each individual, the DPSleep pipeline yielded an estimate of sleep duration (*SleepDuration* in Textbox 1), the time difference between *SleepOnset* and *SleepOffset*. Each day, the individuals also reported the quality of the previous night’s sleep on a Likert scale (Multimedia Appendix 3). Figure S9A of Multimedia Appendix 2 shows the variation in sleep duration across days in each of the 6 individuals from study 1, and their self-reported sleep rating. Missing data excluded from the analysis included data from winter break (the gaps near day 120) and noncompliant data (eg, the wristband was removed or the survey was submitted the following day).

**Figure 4:**
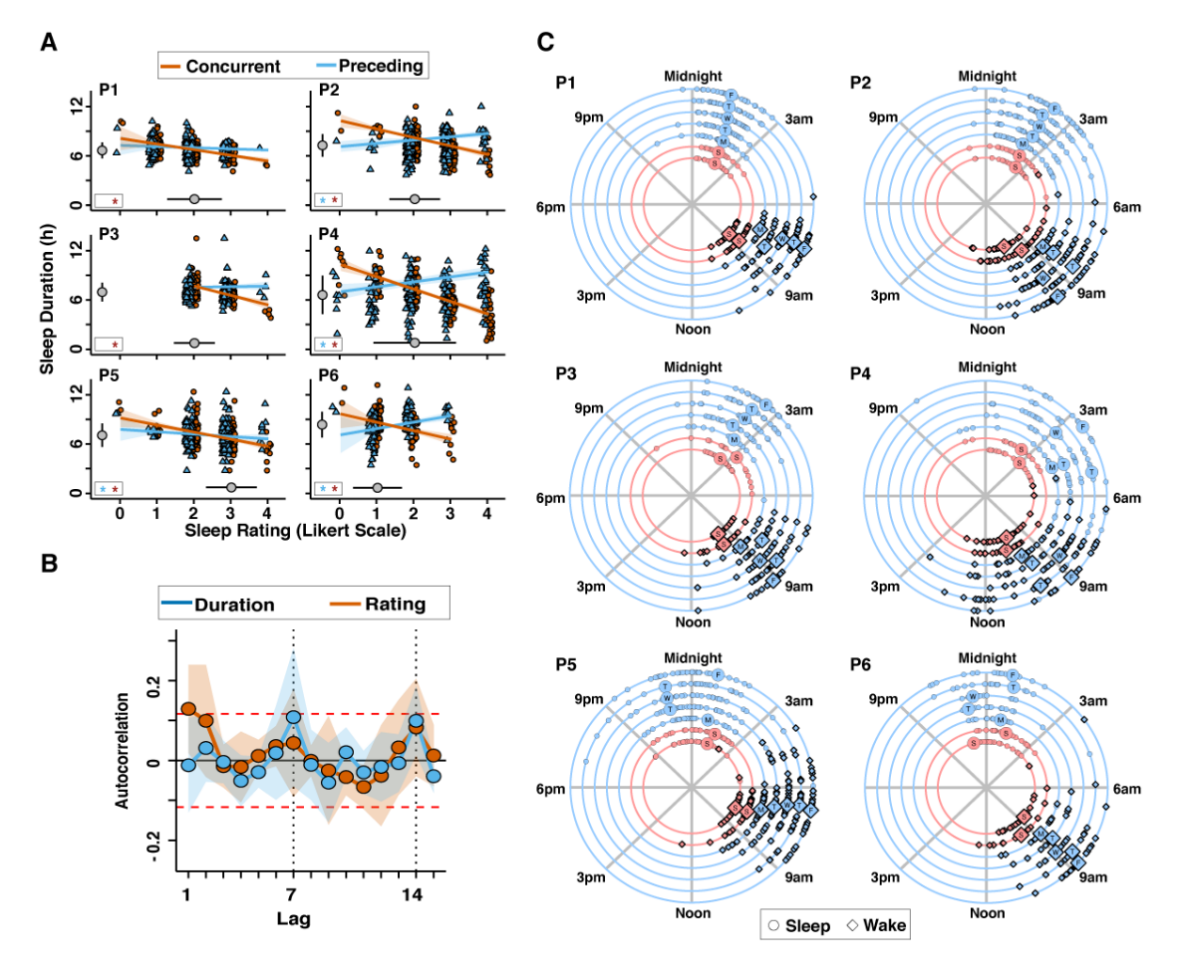
Correlation, autocorrelation, and weekly patterns of sleep rating and sleep duration. (A) Individual-level linear model applied to each subject shows the association between self-report sleep rating and actigraphy-based sleep duration. In each case, the self-report sleep rating negatively predicted sleep duration when the sleep rating targeted the night of sleep (red line and circles). When the sleep rating was shifted to the next day so that the rating no longer matched the night of sleep duration, the relationship between sleep rating and sleep duration in most participants showed either no relation (P1 and P3) or a small positive relation, perhaps a form of sleep rebound (P2, P4, and P6; blue triangles and lines). P5 showed negative association for both nights. The larger gray circles to the left of each plot show the mean (SD) sleep duration across the study for each participant. The larger gray circles below each plot show the most frequent mode (SD) sleep rating across the study for each participant. The bottom-left box in each panel indicates which associations are significant (*P*<.05). (B) The autocorrelation, or the correlation of each variable’s time course with itself at varying lags, is plotted for actigraphy-based sleep duration (blue) and self-report sleep rating (red) averaged across the 6 participants of study 1. Shading illustrates the SE of the mean. Increased autocorrelation values at lags of 7 and 14 days indicate that sleep duration and sleep rating for a given weekly night (eg, Friday or Saturday) are more similar to the same weekly nights on different weeks than to the immediately adjacent nights falling on different weekdays. Although autocorrelation is generally weak, there is a time-dependent structure within the data that should be considered when the data are modeled. (C) Sleep onset and offset (wake) times show differences depending on the day of the week. Each participant’s sleep onset (circles) and offset (diamonds) times are displayed (large circles or diamonds =medians) on a 24-hour clock format. Between-subject sleep onset and offset times are variable, as well are weekday patterns within the same individuals. These structured patterns, which can vary from participant to participant, should be considered when the data are modeled.

An individual-level linear model analysis was used to evaluate the longitudinal association between self-reported sleep quality (for both concurrent night and nights preceding) as the predictor and actigraphy-based sleep duration as the outcome. There was a significant correlation between the concurrent night's self-reported sleep rating and the actigraphy-measured sleep duration in each of the 6 participants (Figure 4A): short sleep duration predicts self-reported ratings of poor sleep. The observation that sleep rating and sleep duration measures track one another in healthy young individuals provides evidence of validity. No correlation or a weakly positive correlation was observed for the preceding night.

### Sleep Duration and Sleep Ratings Show Structured Weekly Variation

One of the outcomes of accurate estimation of major Sleep Episodes in individuals is the ability to investigate nonstationary effects that fluctuate with the weekly schedule, holidays, and academic calendar. An autocorrelation analysis was performed to evaluate the weekly variation. Actigraphy-based sleep duration showed almost no lag effect, such that one night’s duration showed little association with the next (Figure 4B). Interestingly, there was a notable lag effect on days 7 and 14, suggesting a strong autocorrelation between the same days of the week. Self-report sleep rating shows similar lag day 7 and lag day 14 effects and a temporal autocorrelation at lag days 1 and 2, suggesting a poor or good sleep rating predicted a similar rating on subsequent nights despite no evidence of autocorrelation in the actigraphy-based sleep duration.

When data were analyzed by day of the week (Figure S9B of Multimedia Appendix 2), most participants showed relative stability in their sleep duration. We used a circle plot to show the sleep onset and offset distributions for each participant separately on weekdays (Figure 4C). Each point indicates the beginning or end of a major Sleep Episode and their medians. Although there were participants with stable average sleep onset (P1) and offset (P6), the plots confirm different effects of weekdays for different participants, such as the effect of the weekend from Friday to Monday on late sleep in P5; the effect of Monday, Tuesday, and Thursday on very late sleep in P4; and the effect of Monday, Tuesday, and Thursday on earlier wake up in P2, that is, specific structured sleep patterns are highly idiosyncratic to individuals. The daily caffeine consumption was also assessed. Figure S10A of Multimedia Appendix 2 shows the variation in caffeine consumption ranging from 0 (none) to 4 (5+ drinks) for each of the 6 participants in study 1; Figure S10B of Multimedia Appendix 2 shows the weekly variation; Figure S11 of Multimedia Appendix 2 shows the individual-level linear model for each participant modeling the relationship between caffeine consumption and sleep duration. Although the effect is quantitatively small, 4 of the 6 participants show a statistically significant relationship such that more caffeine consumption predicts shorter sleep duration for the upcoming night.

### Example Use Case: Sleep Patterns Show State Variation in Severe Mental Illness

To explore the feasibility and utility of sleep measures from extensive longitudinal assessments in patients, data from 2 individuals managing severe mental illness from study 2 were analyzed using the DPSleep pipeline. Individuals participated for 543 and 309 days, with 89.1% (484/543) and 59.2% (183/309) of completed days of data obtained after data loss due to missingness and quality control, respectively.

**Figure 5:**
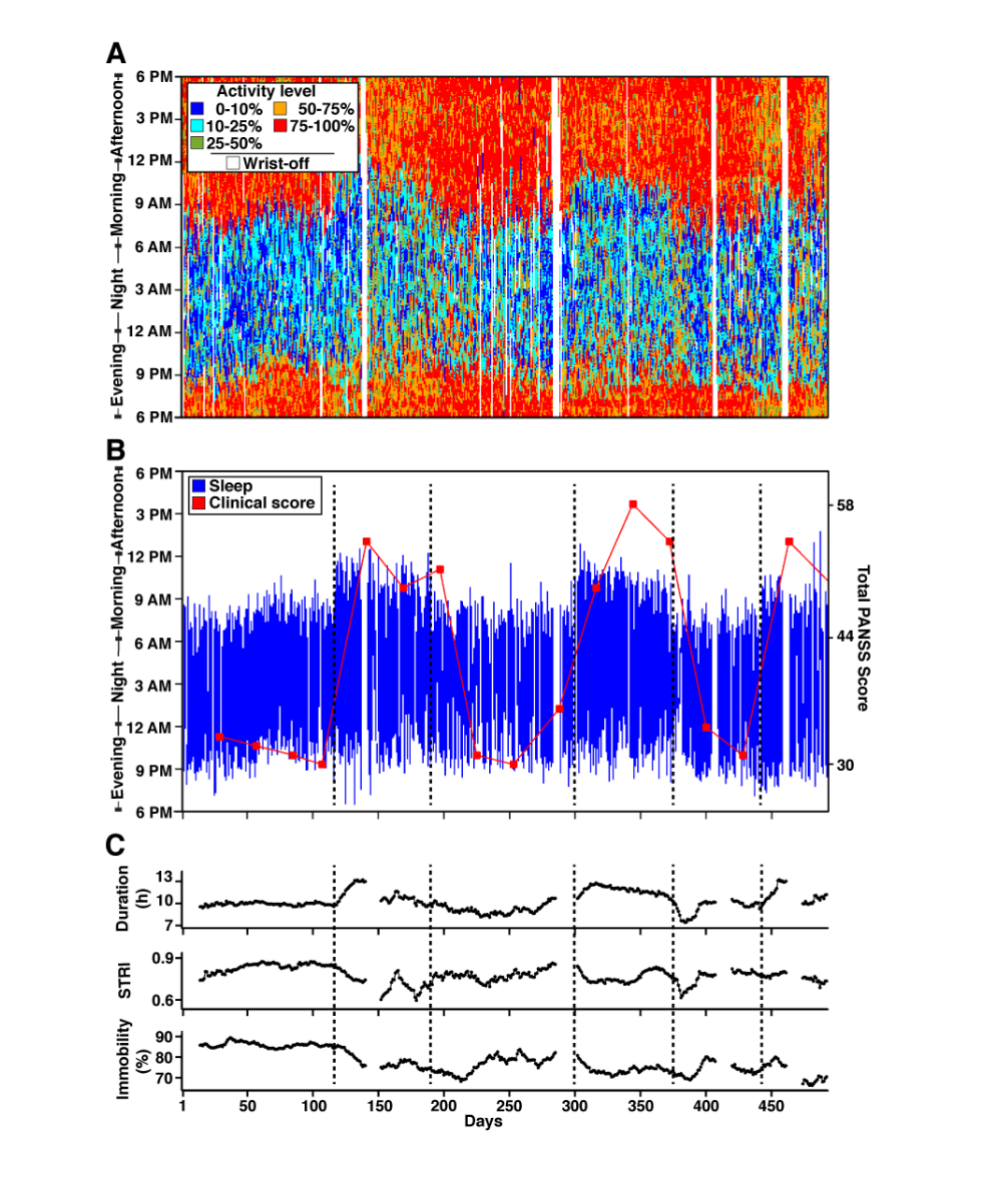
Example longitudinal sleep pattern over 500 days in a patient with severe mental illness. Three panels display longitudinal activity score, Sleep Episode estimates, and clinical severity score and quantitative metrics derived from the actigraphy for P11 of study 2. (A) The top panel displays the continuous activity scores colored by the threshold for each day. (B) The middle panel displays the time zone–adjusted and fully quality-controlled estimates of the Sleep Episodes with the severity of the clinical score overlaid by a red line. The clinical score reflects the total score on the Positive and Negative Syndrome Scale. (C) Temporally smoothed (14-day backward moving average) estimates of three activity-based measures are plotted: the estimated sleep duration (labeled Duration), the Sleep Timing Regularity Index, and the SleepImmobility percentage (labeled Immobility). Gaps in the plots reflect missing days; if more than 2 days were missing, the temporal average that would include those days is absent. Clear state changes in the sleep patterns can be observed (demarcated by dashed black lines in B) that are temporally coincident with negative changes in the clinical score. PANSS: Positive and Negative Syndrome Scale; STRI: Sleep Timing Regularity Index.

Figure 5 illustrates the data from P11, who is managing mood fluctuations originally diagnosed with bipolar disorder. Of interest are the slowly changing sleep patterns that can be immediately visualized in the activity score plots (Figure 5A). Two separate features of the data are interesting and require distinct measures for quantification. The first is that the shifts to low-level activity, indicative of sleep, begin earlier and end considerably later across two long episodes that begin near day 110 and day 300. The reduced activity scores extended until noon on many days. The change in sleep onset and sleep offset and increase in sleep duration was quantified with a 14-previous-day sliding window with less than three missing value tolerance in the DPSleep output shown in Figure 5C. The Sleep Timing Regularity Index (STRI) was also calculated as a 0-1 similarity index for 24-hour sleep and wake minutes of every day compared with an assumed day with the averaged sleep onset and offset. The STRI is a modified version of the Sleep Regularity Index (SRI) introduced by Phillips et al [2]. The SRI "calculates the percentage probability of an individual being in the same state (asleep vs awake) at any 2 minutes 24 hours apart", thus focusing on day-to-day, circadian fluctuations in sleep. The STRI, our modified version of the SRI, compares each study day with each participant's *average sleep day*.

Computing a participant’s average sleep day involves several steps to select the minutes from each day during which a participant is most frequently asleep such that the total duration is equal to the participant's average daily sleep duration. The daily STRI is then computed by comparing each daily Sleep Episode with the average Sleep Episode at the minute level and computing the proportion of minutes for which these two Sleep Episodes matched in sleep. This value, demonstrated in Figure 5C with a backward 14-day sliding window, shows noticeable drops that roughly occur at the beginning of the period of longer sleep intervals. Data with more than three missing values during the sliding window were considered missing.

What is further notable is that the major Sleep Episodes include higher activity score periods than during typical sleep, suggesting interrupted and inconsistent sleep. This feature is picked up in the derived measures of *SleepImmobility* percentage, defined in Textbox 1 as the percentage of *ImmobileMinutes* in the major Sleep Episode illustrated as similar sliding window values in Figure 5C. Adopting a previously described quantitative framing of Immobility [34], the *ImmobileMinutes* are defined in Textbox 1 as minutes with lower than a cut-off threshold (40th percentile activity here), which is the threshold to show the activity level in Figure S3 of Multimedia Appendix 2 (see the *Methods* section). To further illustrate the use of these data, the clinical severity of illness, measured using the Positive and Negative Syndrome Scale Total Score, is shown overlaid on top of the major Sleep Episode in Figure 5B. The periods of sleep disruption and extended sleep offsets correspond to the periods of high illness severity.

In addition, Figure S12 of Multimedia Appendix 2 illustrates data from P12, who lives with psychosis associated with schizophrenia. The activity scores displayed irregular and slowly drifting patterns, including a shift to low activity episodes that occurred later in the day around day 55 and then an earlier shift beginning near day 70 (Figure S12A of Multimedia Appendix 2). The period of most severe illness symptoms occurred near the most irregular sleep periods after day 75. It is to note that this individual has generally poorer clinical scores (Figure S12B of Multimedia Appendix 2) as compared with P11 and a generally lower STRI (Figure S12C of Multimedia Appendix 2). These data illustrate the complexity and richness of information that can be obtained through extensive longitudinal analysis of the actigraphy data and how different individuals can be from one another.

### Example Use Case: Sleep Patterns Related to Deep Dynamic Phenotyping

**Figure 6:**
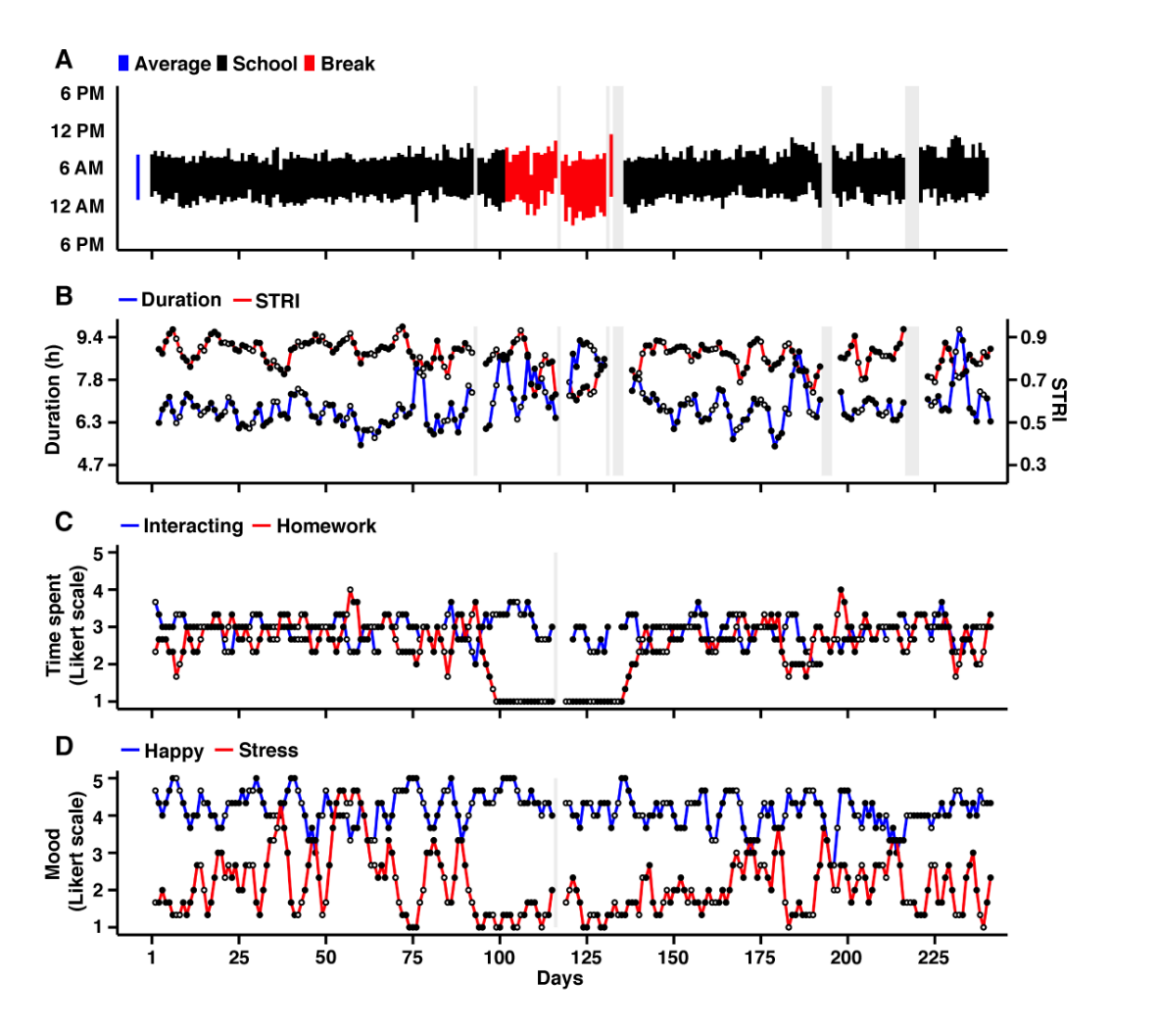
Example year in the life of college student P1. Four panels display multimodality longitudinal data from the full academic year in a college undergraduate (P1 of study 1). (A) Actigraphy-based estimates of the Sleep Episode are displayed, colored by academic period (black=academic school year; red=winter break). Missing data with gray backgrounds reflect missing (wrist-off) or quality removed data (eg, on a travel day across time zones). The mean Sleep Episode is shown to the left in blue. (B) Temporally smoothed (3-day backward moving average with no missing tolerance) estimated of the sleep duration (Duration) and Sleep Timing Regularity Index. (C) Temporally smoothed estimates of time spent interacting and time spent doing homework from the self-report questionnaire. (D) Self-report estimates of mood, including happiness (Happy) and stress (Stress). This individual displays stable and regular sleep patterns with periodic deviations. Note the deviations in time spent interacting and time spent doing homework during the break period. STRI: Sleep Timing Regularity Index.

To demonstrate how sleep measures can be combined with additional forms of digital phenotyping information, 2 individuals from study 1 are displayed in Figure 6 and Figure S13 of Multimedia Appendix 2. In each plot, the sleep patterns for each individual are illustrated through the course of an academic year, along with the quantified measures of sleep duration and STRI. In addition, self-report measures of social and academic behaviors (time on homework and time interaction) and mood (happy and stress) were displayed aligned to the estimates of the longitudinal Sleep Episodes. The examples illustrate clear dynamics of sleep, including weekend (empty circles) or weekday (filled circles) effects, changes between the active semester and breaks, and intermittent deviations for regular patterns. These data illustrate the potential of using low-burden wearables in combination with smartphone-based digital phenotyping to capture a great deal of information about life rhythms and changes due to environmental demands.

### An Example of Limitations

In showing examples of the utility of the DPSleep processing pipeline and example apps, we wanted to also show, as the last point, a clear example of a limitation. The present pipeline and quality control adjustments make assumptions that have been selected because they work most of the time in the range of contexts for which they were tested. However, real-life situations are complex. Figure S14 of Multimedia Appendix 2 shows an interesting example. In this example, the daily data from Figure S2 of Multimedia Appendix 2 are replotted along with phone use and GPS location data from the same participant of the third example. What is notable is that our original estimate, based only on watch actigraphy data and the button press, combined with the rule used to join episodes of low activity as a continuous major Sleep Episode, likely mischaracterizes this night's true continuous Sleep Episode. The phone data reveal that the nighttime period of activity is followed by missing GPS data, intermittent phone use, and eventual arrival at a new location. This individual most likely woke up early and got on a bus or train. Our automated estimation procedures, as well as our quality control, using only watch actigraphy, mischaracterizes this Sleep Episode.

## Discussion

### Principal Findings

This work describes and demonstrates the utility of an open-source, longitudinal sleep-analysis platform called DPSleep. The platform was applied to two cohorts of participants that possessed extended data over months to years and included clinically healthy undergraduates and outpatients living with severe mental illness. The goal of these diverse explorations was to validate the approach and demonstrate its utility across multiple real-world participant cohorts. The results revealed that the approach captured the major Sleep Episode and detected dynamic patterns of sleep behavior, including in individuals presenting with episodic clinical illness (eg, Figure 5 and Figure S12 of Multimedia Appendix 2).

Several previous studies have investigated the validity and accuracy of wrist-worn actigraphy devices to estimate sleep parameters and compare them against gold standard PSG-derived measures and self-reported sleep quality [49]. DPSleep uses frequency-based analysis and begins with high-frequency raw data from the wrist-worn accelerometer. The high precision of the sampling frequency and the continuity of the collected data in our samples provided ideal data sets for frequency-based analysis, a robust and efficient tool to analyze the activity of the individual that complements alternative procedures such as zero crossing mode, time above threshold, or digital integration mode [19]. Frequency-based analysis inherently and automatically discounts the alternating gravity effect on different axes, without the need to eliminate the amplitude of the signal [50]; at the same time, it disentangles the natural high-frequency shakes of the body from the actual repositioning dynamic, which is the focus of sleep research. As explained earlier, the raw accelerometer data contain the gravity component offset that can be systematically removed in the frequency analysis.

A challenge with our approach was related to participant burden, as the participants had to visit the lab every 4-5 weeks to get a refreshed battery and download data. An alternative solution to attenuate the burden on subjects is to use wireless data transfer and cloud storage; however, this approach has its own limitations, based on the wireless storage size and battery usage in addition to data security concerns. We expect advances in widely available commercial technologies to gradually alleviate existing challenges. Another challenge was the time changes caused by travel or standard or daylight saving switches during the study. To reduce the uncertainty around participants’ true sleep behavior when the time was shifted, days with time transitions were removed and considered missing data, although future steps could leverage the phone-based GPS data to exclude the relocating epochs from sleep and adjust the data on those days with more confidence.

In our study, self-reported sleep quality showed a strong correlation with the activity-based sleep duration measure; therefore, the focus of our sleep algorithm development was mainly to optimize this parameter, while building a platform to explore other relevant sleep-related parameters in future work. Despite existing sleep detection algorithms that look for 5-15 low activity minutes [50,51], DPSleep starts with a structural analysis of the daily activity to first detect the large episode of the day with the lowest average activity, and then the edges of the episode are adjusted using smaller moving windows and heuristic rules. This approach is an efficient solution to eliminate the need for any kind of sleep diary or ambient light assumptions because the former is not usually available and accurate, and the latter could be misleading because the wristband can be blocked by a long sleeve at any time of the day. Moreover, this approach distinguishes short naps or inactive periods during the day from the long, continuous, and disrupted Sleep Episodes without any assumptions about sleep time. Structural analysis of the activity scores during the whole study, using the individual's statistics and moving average windows with different sizes, suggests the most likely episode for the individual’s sleep during the day. In addition to moving average windows to automatically detect the major Sleep Episode, DPSleep provides a helpful tool for investigators to decide, with reasonable confidence, about idiosyncratic sleep behaviors such as no sleep or very short Sleep Episodes. Additional button presses, if available, and adjacent activities are used to tune this episode and connect smaller pieces to shape the entire Sleep Episode.

### Caveats and Limitations

As illustrated in Figure S14 of Multimedia Appendix 2, our approach can make mistakes. Although we illustrate how inexpensive and easy-to-obtain actigraphy data can be analyzed to estimate the major Sleep Episodes and dynamic patterns over many days, the real world is messy. Atypical behavioral patterns (eg, many short naps without a clear extended primary Sleep Episode) and behaviors that yield extreme accelerometer readings are challenging for our approach. Specifically, using our methods, actigraphy-based sleep detection is not appropriate for measuring sleep when an individual is on a shaking platform such as a plane, train, or bus. This is an unavoidable situation in longitudinal studies; however, as the on-plane sleep effect was not the focus of our study, and those days were very few compared with the days of the entire study, we were able to detect those days using GPS data and eliminate them from the results. To solve the continuous shaking challenge in similar situations or in studies on individuals with Parkinson disorder, sleep detection devices based on ambulatory circadian monitoring are recommended [28]. More broadly, as actigraphy research grows and repositories of annotated data from common and less common activities become available, machine learning techniques can be leveraged to further refine the present approach (and others) handle a wider variety of situations. The validation and exploration of DPSleep, as illustrated in these initial explorations, provides a tool that can be used today and continues to be refined and expanded through its open-source release. Future directions include examining whether the association between mood and actigraphy-based sleep and activities can serve as a biomarker of psychiatric illness, and eventually use these objective measures to predict clinical patients’ mood fluctuations.

## Appendix

Multimedia Appendix 1 Self-reported questionnaire.

Multimedia Appendix 2 Extended figures.

Multimedia Appendix 3 Quality control instruction.

## Abbreviations

DSM: Diagnostic and Statistical Manual of Mental Disorders
PSG: polysomnography
RMS: root mean square
SRI: Sleep Regularity Index
STRI: Sleep Timing Regularity Index
