# Supplementary material for: Open-source Longitudinal Sleep Analysis From Accelerometer Data (DPSleep): Algorithm Development and Validation
Source: JMIR Mhealth Uhealth. 2021 Oct 6;9(10):e29849. doi: 10.2196/29849 (PMC8529474; doi:10.2196/29849)
Supplement: Multimedia Appendix 1 [file mhealth_v9i10e29849_app1.docx]

**Appendix I: Self-Report Questionnaire**

**Sleep**

How did you sleep last night?

1) Terribly: little or no sleep
2) Not so well: got some sleep but not enough
3) Sufficient: got enough sleep to function
4) Good: got a solid night’s sleep and felt well-rested
5) Exceptional: one of my best nights of sleep

**Caffeine**

In the past 24 hours how much caffeine (e.g., 8oz of coffee, tea, or soft drink) did you consume?

1) None
2) One drink
3) Two drinks
4) Three or four drinks
5) Five or more drinks

**Interacting**

How much of your waking time did you spend interacting with others over the past 24 hours?

1) Very little of my time (0-20%)

2) Some of my time (21-40%)
3) About half of my time (41-60%)
4) Most of my time (61-80%)

5) Almost all of my time (81-100%)

**Homework**

How much of your waking time did you spend studying or doing homework over the past 24 hours?

1) Very little of my time (0-20%)

2) Some of my time (21-40%)
3) About half of my time (41-60%)
4) Most of my time (61-80%)

5) Almost all of my time (81-100%)

**Happy**

How much did you feel happy over the past 24 hours?

1) Very slightly or not at all
2) A little
3) Moderately
4) Quite a bit
5) Extremely

**Stress**

How much did you feel stressed over the past 24 hours?

1) Very slightly or not at all
2) A little
3) Moderately
4) Quite a bit
5) Extremely
