# Supplementary material for: Open-source Longitudinal Sleep Analysis From Accelerometer Data (DPSleep): Algorithm Development and Validation
Source: JMIR Mhealth Uhealth. 2021 Oct 6;9(10):e29849. doi: 10.2196/29849 (PMC8529474; doi:10.2196/29849)
Supplement: Multimedia Appendix 2 [file mhealth_v9i10e29849_app2.docx]

**Figure S1. Three-axis accelerometer data for a day of data.** The raw acceleration signal along three axes sampled here at 30 Hz and scaled to 9.8 m/s^2^ gravity intuitively shows the change in the dynamics of the signal, including the Sleep Episode from approximately 12 AM to 7 AM.

**Figure S2. Examples of daily sleep reports.** Each panel A-C displays a sleep report for a single 24-hour day of a participant. All data are from the watch. For each panel, the top row displays the activity level over time as the power of one minute (*Activity Level*). The activity score is shown in color in the 2nd row, with the low activity periods quite apparent in blue (*Activity Score)*. The third row (*Sleep*) displays the actual estimates of the Sleep Episode derived fully automatically from the activity scores and the button presses (when available). The initial provisional estimate of the Sleep Episode (called Algorithm) is displayed in yellow, with the automatically expanded Sleep Episode above it. The Sleep Episode is the main estimate used for the calculation of *SleepOnset*, *SleepOffset*, and *SleepDuration*. The Bedrest Episode is also shown, which extends beyond the Sleep Episode to include adjacent medium activity periods. The final row (*Light*) shows the light levels.

**Figure S3. Example of a daily sleep report that includes phone use and GPS data.** Data for a single 24-hour are displayed similar to Figure 3 with additional data obtained from the smartphone of the individual. The first four rows are identical to Figure 3. The fifth row (*Phone*) displays lock-unlock phone events. The sixth row (*Acceleration*) displays data derived from the accelerometers within the phone. The final row (*GPS*) shows the clustered frequently visited locations of the individual relative to their estimated home location (dark gray), with different colors showing different distances from home. Missing GPS data are shown in white and light gray indicates the available coordinates that are not among the frequently visited locations by the individual.

**Figure S4. Effects of manual quality control adjustment on Sleep Duration.** The Raw Sleep Duration estimates for each of the 6 participants of study 1 are plotted against the Corrected Sleep Duration estimates after quality control adjustment.

**Figure S5. Examples of manual adjustment of the Sleep Episode.** Two examples of adjustments to the Sleep Episode are illustrated. The adjusted episode is noted by the black line with marked ends plotted on top of the automatically generated Sleep Episode (green bar) and Bedrest Episode (blue bar). (A) A case where the automated process identified a short sleep adjacent to the main Sleep Episode. The two were concatenated into one extended Sleep Episode. (B) A case where the stillness during sleep appears to fall below the threshold and gets erroneously labeled as a wrist-off event (white, missing data). However, the intermittent movements suggest the watch is being worn. Manual adjustments only used the data from the watch actigraphy device.

**Figure S6. Longitudinal Sleep Episode estimates in P2 in relation to phone use**. Longitudinal Activity Score, Sleep Episode estimates, and phone use data for 257 days are plotted in the same format as in Figure 2. Day 2 is near to the beginning of the semester. Winter break falls near days 112 to 143. An interesting feature in this individual is the extended wrist-off periods from Days 194 to 201. As can be seen in panel C, these days in their entirety are excluded from the final analysis. Another interesting feature in this individual is the gradual shift to a later Sleep Episode from Day 1 to about Day 25, indicated with the red asterisk in B and C. The later Sleep Episode is then maintained for much of the semester. There is minimal phone use during the Sleep Episodes, consistent with this individual not using their phone during the nighttime. In contrast to P1, Phone Status events begin almost immediately following waking, suggesting that the individual starts using her or his phone as soon as sleep ends.

**Figure S7. Longitudinal Sleep Episode estimates in P3 in relation to phone use**. Longitudinal Activity Score and Sleep Episode estimates, and phone use data for 258 days are plotted in the same format as in Figure 2. Day 4 is near to the beginning of the semester. Winter break falls near days 114 to 146. Similar to P2, this individual shows a gradual shift to a later Sleep Episode from Day 1 to about Day 25, indicated with the red asterisk in B and C. In this individual, phone use often ends well before the estimated Sleep Episode begins. In addition, phone use shows a structured pattern on wakening with often-recorded acceleration events at 9 AM, then a gap, and then another bout of events at 10 AM.

**Figure S8. Two-Day column formatted Longitudinal Activity Score and Sleep Episodes.** Color-coded Activity Score (A) and the estimated Sleep Episode (B) are presented in a format that is standard in the sleep medicine community. In this format, the days of the study are presented as the rows from top to bottom and every row contains the data of two consecutive days; the first column is the original day and the second column is the next day both presented from 12 AM to 12 AM. The format allows the investigators to focus on the study nights with the vertical blue band in the middle of the plot, without missing any information before and after the night. This individual (P1) Sleep Episodes are shifted to the right of the center because their sleep onset time is typically after 1 AM.


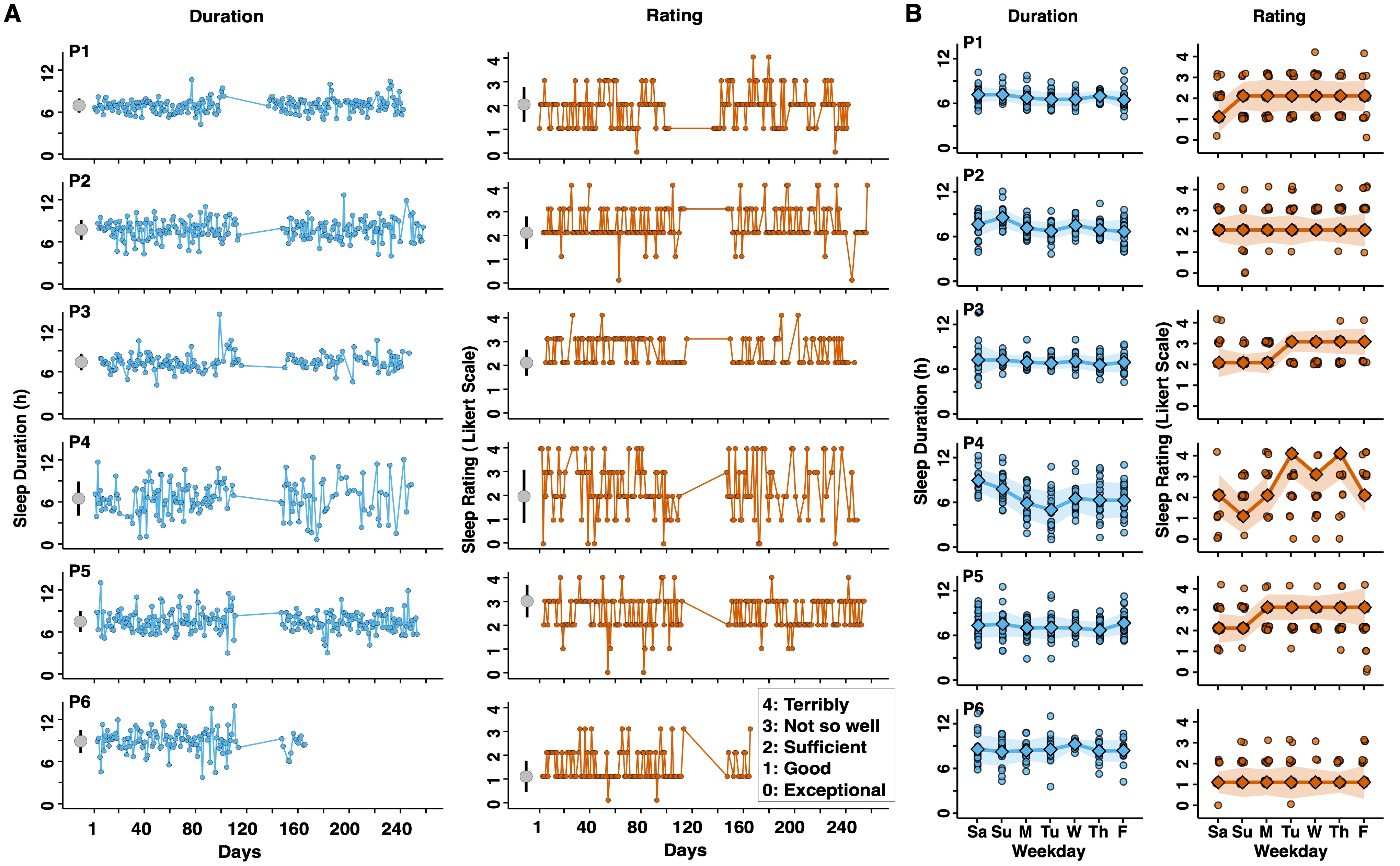


**Figure S9. Longitudinal Behavior and Weekly Patterns of Sleep Duration and Sleep Rating.** (A) The time course of each participant's objective Sleep Duration (in minutes) as measured by actigraphy is plotted (left column) next to the same participant's self-report Sleep Rating (right column; 5-point Likert scale; 0=Exceptional, 4=Terribly). Data from winter break and non-compliant data (e.g., the watch was removed, or the survey was submitted the following day) are excluded; hence there are gaps in the data, including a large gap around day 120 (winter break). P6 traveled out of the country at the beginning of the second semester, so collecting the accelerometer data from them was not feasible at that time. The gray circles to the left of each time course show the mean (SD) Sleep Duration and most frequent (mode (SD)) Sleep Rating for each individual. (B) Sleep Duration and Sleep Rating are plotted separately for each weekday in each of the 6 participants of study 1 that vary depending on the day of the week for some participants. While some participants show relative stability in Sleep Duration (P1, P3, P5), others show strong effects of weekday (P2, P4). The self-report Sleep Rating is also variable with a tendency for the nights before the weekend days to be rated better (Saturday, Sa; Sunday, Su). Circles representing data points for Sleep Rating are jittered for visualization. Means (for Sleep Duration) and medians (for Sleep Rating) are shown by enlarged symbols with the shaded surround representing the standard error of the estimate.


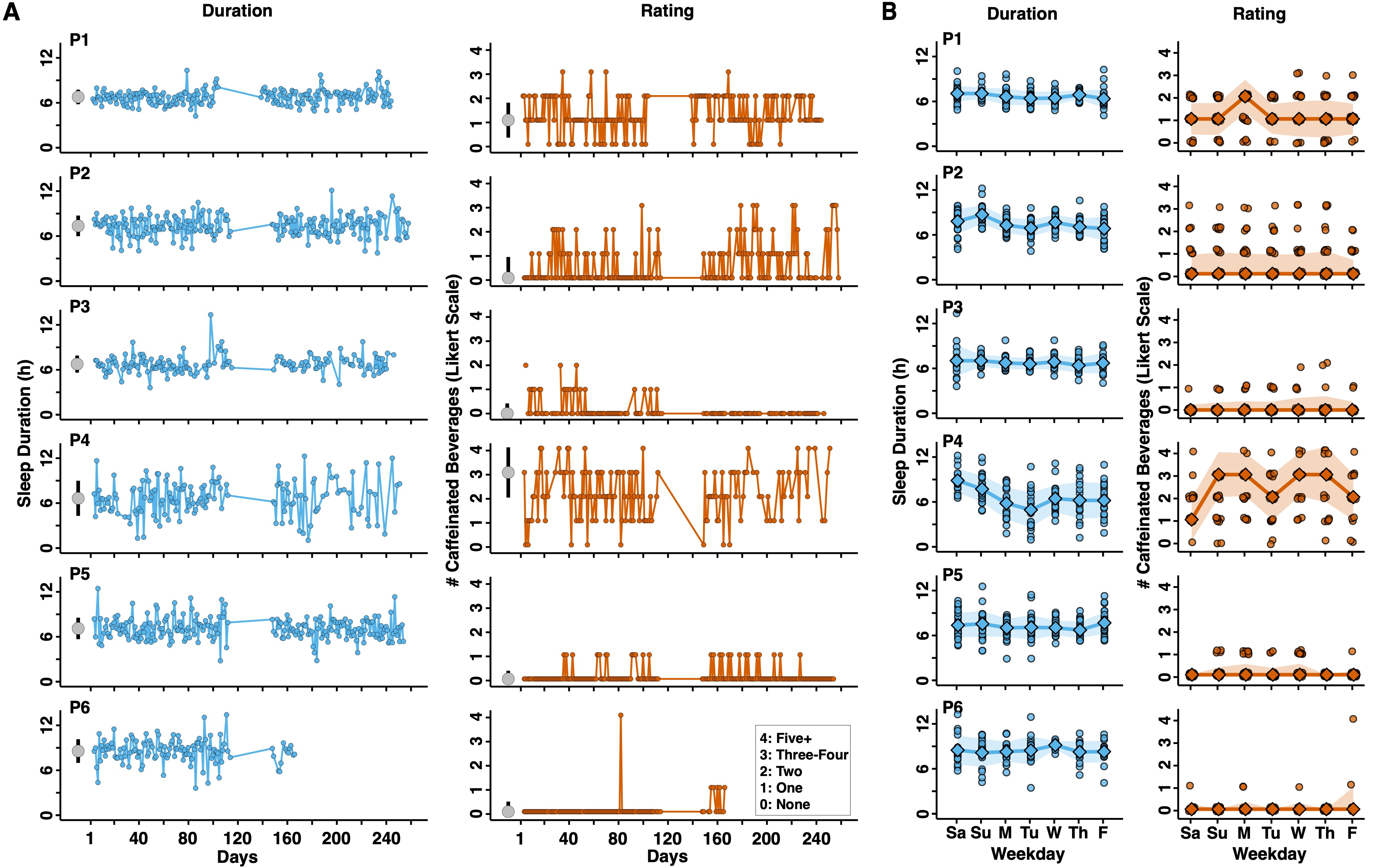


**Figure S10.** **Longitudinal Behavior and Weekly Structure of Sleep Duration and caffeine consumption.** (A) The time course of each participant's actigraphy-based Sleep Duration (in minutes) is plotted (left column) next to the same participant's self-report caffeine consumption (right column; 5-point Likert scale; 0=None, 4=Five+). The plot format is similar to Figure S9. Even in this small sample, the between-subject and within-subject variability in caffeine consumption is notable, with certain participants drinking caffeinated beverages often and variably across days (P1) and others consuming minimal caffeine on average with exceptions (P6). (B) Sleep Duration and caffeine consumption plotted separately for each weekday in each of the 6 participants of study 1 show variation depending on the day of the week. Circles representing data points for # Caffeinated Beverages are jittered for visualization. Means (Sleep Rating) and medians (# Caffeinated Beverages) are shown by enlarged symbols with the shaded surround representing the standard error of the estimate.


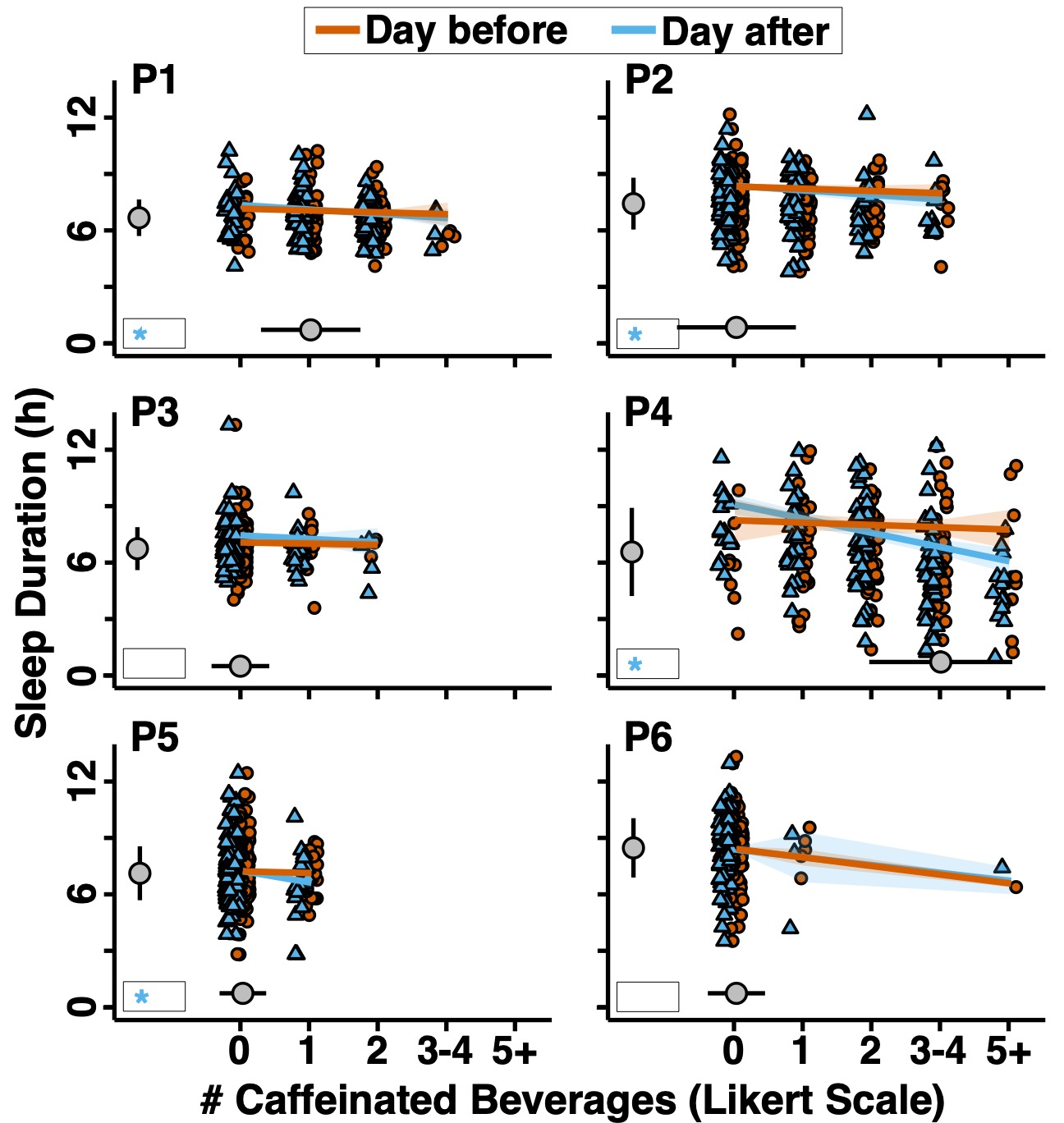


**Figure S11. Self-report caffeine consumption predicts Sleep Duration**. The individual-level linear model framework shows individualized associations between Sleep Duration and self-report ratings of the number of Caffeinated Beverages consumed. Data are plotted similar to Figure 4 part A and illustrate significant associations in certain participants (P1, P2, P4, P5) but not others (P3, P6).

**Figure S12. Example longitudinal sleep pattern over 200 days in a patient with severe mental illness**. Using the same format as Figure 5, data from P12 of study 2 are plotted. Note the consistently irregular and fragmented sleep patterns that are pervasive throughout the course of the measurements. These data illustrate the feasibility of collecting high-quality continuous data using passive actigraphy in individuals with severe mental illness.

**Figure S13. Example year in the life of college student P5**. Using the same format as Figure 6, four panels display multi-modality longitudinal data from the full academic year in a college undergraduate (P5 of study 1). This individual shows highly irregular sleep during the academic year and during the break. Note that in addition to the general variation in sleep patterns and mood, there are specific instances of extreme variations that are reflected in Sleep (e.g., Day 144 in B) and Stress (D).

**Figure S14. Example of an erroneous estimate of the Sleep Episode.** Plots of a night from study 1 illustrate that actigraphy-based sleep estimation is imperfect. The top four rows represent the watch-based actigraphy information as well as our present procedure's estimates of the Sleep Episode (from Figure S2). The bottom three rows show the phone use data for this night plotted in an aligned format. Note that the 'active' period near 5 AM is followed by periods of phone use and acceleration, and then a GPS-estimated change in location (see pink location estimate starting at 11 AM). This participant likely got out of bed and went into a car or bus, sitting relatively motionless for several hours, until she or he arrived at a new location. Our procedures, which only use the watch data, mischaracterized this Sleep Episode. This complex night is a reminder that real-world behavior is complex and that the assumptions of the approach are imperfect for all situations.
