# Supplementary material for: Open-source Longitudinal Sleep Analysis From Accelerometer Data (DPSleep): Algorithm Development and Validation
Source: JMIR Mhealth Uhealth. 2021 Oct 6;9(10):e29849. doi: 10.2196/29849 (PMC8529474; doi:10.2196/29849)
Supplement: Multimedia Appendix 3 [file mhealth_v9i10e29849_app3.docx]

**Appendix II: Quality Control Instruction**

On the Activity score plot: The activity level in each minute is color-coded so that Cyan and Blue are the lowest activity minutes and Orange and Red are the minutes with the highest activity. Green is the medium activity. The Sleep Episode is almost continuous appearance of Blue and/or Cyan (lowest activity).

On the Sleep plot:

- **Light yellow box** is the initial provisional estimate of the Sleep Episode automatically detected by the algorithm (called Algorithm on the Sleep plot).
- **Green box** is the Sleep Episode estimation when the sleep edges are adjusted to:

1. Button Press Markers: if the marker(s) exist(s) and is(are) less than 60 minutes inside the sleep box detected by the algorithm; otherwise:
2. When the edge of the Algorithm box is within the Cyan/ Blue block: Choose the first minute out of the Algorithm box that does not have the lowest activity. It can be Green, Orange or Red. In the other word, extend the Sleep Episode to the edge of the continuous Blue/Cyan block.
3. When the edge of the Algorithm box is out of the Cyan/ Blue block: Choose the first minute inside the Algorithm box that has the lowest activity. In other words, shrink the Sleep Episode to the edge of the continuous Blue/Cyan block.

- **Blue box** is the Bedrest Episode estimation which is set to:

1. Button Press Markers: if the marker(s) exist(s) and is(are) less than 60 minutes outside the sleep box detected by the algorithm; otherwise:
2. When the edge of the Algorithm box is within a Cyan/ Blue block: Choose the first minute out of the Algorithm box that has high activity. It can be Orange or Red, but not Green. In other words, extend the Bedrest Episode to the first high active minute.
3. When the edge of the Algorithm box is out of the Cyan/ Blue block: Choose the first minute outside the Sleep Episode box that has high activity (Orange or Red).

**Notice:** In case there are short intervals of detected wrist-off minutes (White) during sleep, which are not larger than 150 minutes consider them as very low activity minutes (Blue). These are the very low activity minutes occasionally happening during the Sleep Episode that the sliding window erroneously tags as watch-off, and the activity of the minutes around them shows that the watch is actually not off the wrist and they are part of the Sleep Episode.

**Notice:** If there is no Sleep Episode that contains more than 45 continuous lowest activity minutes, report No Sleep.

**Notice:** The general rule for the Sleep Episode is to choose the longest episode that contains one or more low activity epochs, and multiple epochs can be connected to each other with no more than 90-min active epochs (bouts) in between, and choose the rest as Nap Periods. If the longest epoch is after 12PM and there is a shorter epoch before, choose the shorter one as Sleep Episode.
